# Influence of hypophysectomy, ovariectomy and gonadectomy on postoperative hypersensitivity in rats

**DOI:** 10.15761/GAPM.1000145

**Published:** 2016-05-20

**Authors:** Dustin P. Green, Mayur J. Patil, Armen N. Akopian

**Affiliations:** 1 Department of Pharmacology, UT Health Science Center, San Antonio, TX, USA; 2 Department of Neuroscience, Johns Hopkins University, School of Medicine, Baltimore, USA; 3 Department of Endodontics, UT Health Science Center, San Antonio, TX, USA

**Keywords:** hypophysectomy, ovariectomy, surgical procedure, pain

## Abstract

Surgical procedures lead to profound and sustained (up to 1–2 weeks) activation of the pituitary gland, resulting in changes in endocrine function. Questions remain on whether activation of the pituitary influences the threshold and development time-course of postoperative pain. To address these questions, we evaluated postoperative hypersensitivity in female and male rats with ablated pituitary and gonadal hormone productions via hypophysectomy, ovariectomy and gonadectomy, respectively. Plantar incision, a model of acute postoperative pain, or sham operation was performed on rat hind paws. Hypophysectomy, ovariectomy and gonadectomy were achieved by surgical disconnection of pituitary, ovaries and testicles, respectively. Postoperative thermal and mechanical hypersensitivity were monitored for 7 days post incision. Hypophysectomy on female and male rats produced statistically similar thermal and mechanical postoperative hypersensitivity thresholds and time-courses as compared to intact estrous female and male rats. Moreover, ovariectomy and gonadectomy did not significantly change postoperative hypersensitivity observed in control female and male animals. Our experiments demonstrate that hypophysectomy, ovariectomy and gonadectomy do not significantly impact postoperative hypersensitivity observed in normal female and male animals. These data suggest that surgery-induced changes in the endocrine system via activation of pituitary and subsequently gonadal tissues have little impact on the threshold and development of postoperative pain in female and male rats.

## Introduction

It is well documented that surgical intervention profoundly modulates the endocrine system leading to complex metabolic events [1–3]. Tissue injury and psychological stress associated with surgeries and/or anesthetics used during surgical procedures could be major contributors in modulating the endocrine system [4]. There is evidence that the response of the endocrine system to surgical intervention is mediated through the hypothalamic-pituitary-adrenal (HPA) axis [5]. Thus, major and some minor surgeries usually result in systemic sustained increase of adrenocorticotropin (ACTH), growth hormone (GH) and prolactin (PRL), whereas plasma estradiol and testosterone levels are decreased for up to 1–2 weeks after surgery [1,6–8]. The magnitude of HPA responses vary depending on type and the extent of surgery, as well as patients’ sex and age [5,7,9–12].

Plasma levels of some hormones correlate with severity of postoperative pain [13,14]. A severe catabolic state driven by a prolonged endocrine reaction to surgery may be linked with an increased morbidity and mortality in high-risk adult patients [2]. Further, successful management of postoperative pain restores the normal serum levels of these hormones [10,13]. Systemic elevation of hormones after surgery could stimulate the immune system [9,15,16], which plays a critical role in regulating pain development and threshold [17].

Despite the wealth of data on modulation of the endocrine system by surgical intervention, an important important question remains unanswered. Do pituitary and gonad-produced hormones directly impact the threshold and development of postoperative pain? This critical information could provide major clinical benefits in the diagnosis as well as the management of postoperative in patients undergoing surgical procedures and stress. Here, we will address the question of whether pituitary and gonad-produced hormones contribute to postoperative pain. Pituitary and gonadal hormone productions will be stopped via hypophysectomy (HYPO) in females and males, ovariectomy (OVX) in females and gonadectomy (GDX) in males, respectively. Plantar incision on rat hind paws will be used as a model to study acute postoperative pain. This rodent surgery closely models surgical effects on the endocrine system in humans, leading to elevation of plasma levels for ACTH [18], GH [19] and PRL [20], as well as reduction in gonadal hormones [21,22].

## Materials and methods

### Animals, hypophysectomy, ovariectomy and gonadectomy

All animal experiments conformed to APS’s Guiding Principles in the Care and Use of Vertebrate Animals in Research and Training, and to protocols approved by the University Texas Health Science Center at San Antonio (UTHSCSA) Animal Care and Use Committee (IACUC). We also followed guidelines issued by the National Institutes of Health and the Society for Neuroscience to minimize the number of animals used and their suffering.

Intact and operated adult female and male Sprague-Dawley rats (200–250g, Charles River Laboratories, Wilmington, MA) were housed three per cage under a 12-h light/12-h dark cycle with food and water available ad libitum. Additionally, hypophysectomized animals received water containing 5% dextrose and 0.9% NaCI. Adult female and male HYPO, OVX female and GDX male rats were obtained from Charles River Laboratories (Wilmington, MA). Hypophysectomy, ovariectomy and gonadectomy are achieved by surgical disconnection or removal of pituitary, ovaries and testis, respectively. Gonadal hormones in serum were measured using 17β-estradiol high sensitivity and testosterone ELISA kits (both ENZO). PRL serum levels were measured as previously described using rat Prolactin ELISA kit (Cayman Chemical) [20].

### Acute postoperative pain model

All behavioral experiments were conducted by a blinded observer. Since HYPO and OVX female rats have no regular estrous cycles, surgical procedures on these female rats were conducted without taking into account the estrus phase. The reproductive stage of cycling intact females was determined by vaginal lavage using methods previously described [23]. The plantar incisions in rats were performed as previously described [24]. Briefly, rats were anesthetized using 2% isoflurane. The right hind paw was prepared for incision by application of antiseptic betadine solution. The incision model in rats was created by a 1-cm longitudinal incision, through skin and fascia of the plantar aspect of the foot, beginning 0.5 cm from the proximal edge of the heel and extending toward the toes. Curved forceps were used to elevate longitudinally the underlying the plantaris muscle. A mattress suture of 5-0 nylon on a FS-2 needle (Ethicon, Somerville, NJ) was used to close the incision. Antibiotic neomycin ointment (Bacitracin Zinc Ointment USP, Melville, NY) was applied to the wound and the sutures removed 2 days later. Sham treatment consisted of rats that received anesthesia, antiseptic preparation and topical antibiotic without an incision. Thermal and mechanical hypersensitivity were measured in these animals over a period of 7 days.

### Thermal hypersensitivity

Rats were habituated to the testing environment for at least 30 min prior to testing. Heat nociception was assessed as previously described [25]. In brief, rats were placed on a glass surface with temperature held constant at ≈20°C. Following habituation, thermal withdrawal latencies to a radiant heat beam focused on the plantar area of the hind paw were recorded at each time point (3× measurements at each time point, averaged to obtain the data value used in analyses). In order to prevent tissue damage, the stimulus was terminated after ≈20 sec if the animal did not withdraw the hind paw.

### Mechanical hypersensitivity

After habituation to the testing environment, the baseline readings (three readings per animal) were taken on the right hind paw using the Dynamic Plantar Aesthesiometer (UgoBasile) to record withdrawal thresholds for mechanical stimulation of the plantar area of the hind paw. The instrument applies constant ramp of increasing mechanical pressure to the paw (from 0 to 50 grams over 10 second intervals) and the withdrawal threshold was recorded in grams when the paw was withdrawn.

### Data analyses

GraphPad Prism 5.0 (GraphPad, La Jolla, CA) was used for statistical analyses. The data in Figures were given as mean ± standard error of the mean (SEM), with the value of “n” referring to the number of analyzed animals for each group. All experiments were performed in duplicate with the use of a different set of rats. Differences between groups were assessed by two-way analysis of variance (ANOVA; no matching, regular 2-way ANOVA, not repeated measures) with Bonferroni’s multiple comparison post-hoc tests (where each column was compared to all other columns). Differences between male and female post-operative hyperelgesia were conducted with 3-way ANOVA with sex, rat line (*i.e*.,hypophysectomized, ovariectomized or gonadectomized) and time as factors. A difference was accepted as statistically significant when p<0.05.

## Results

### Hypophysectomy and postoperative hypersensitivity

**Figure 1:**
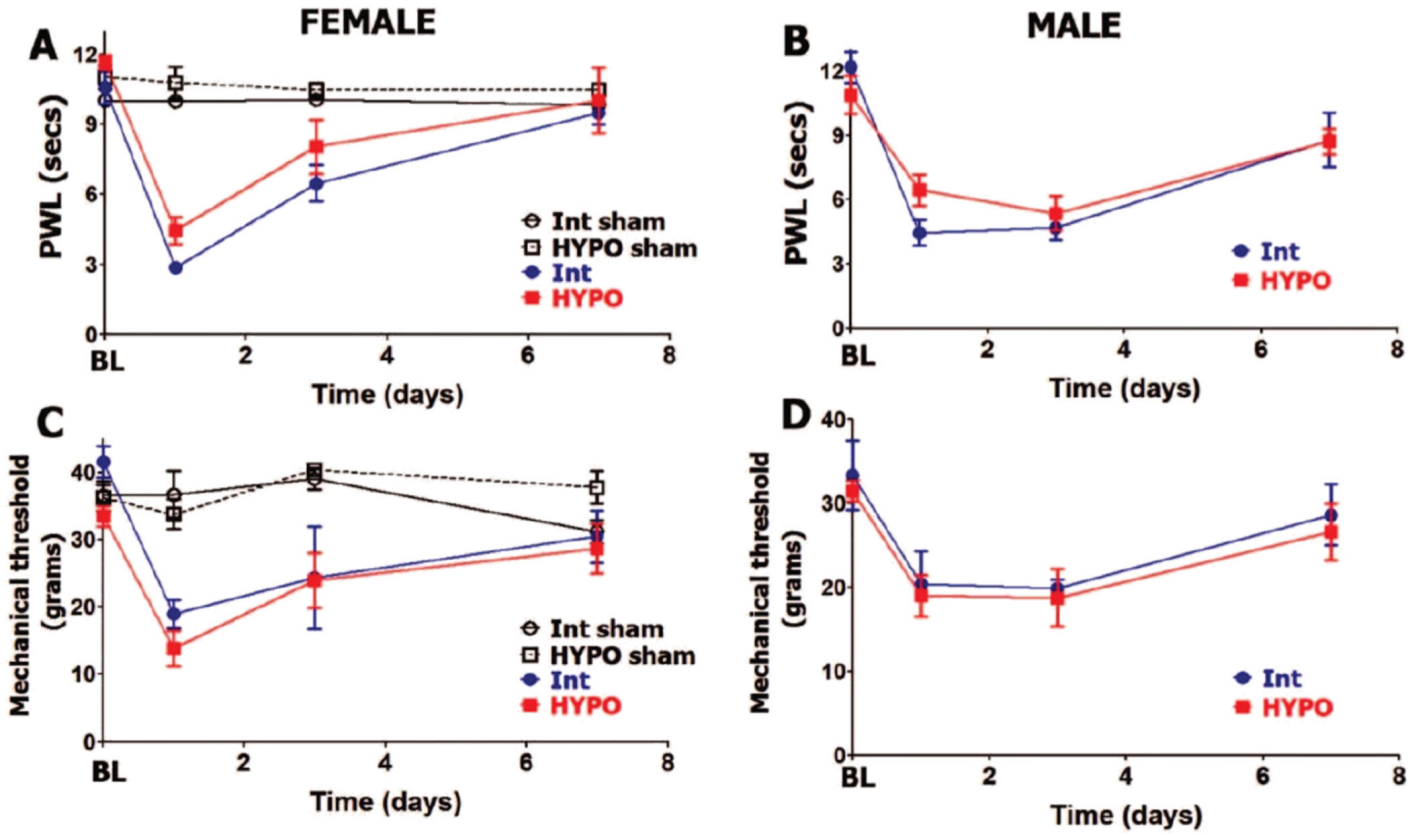
Hypophysectomy and postoperative hypersensitivity in female and male rats Incision-induced thermal hypersensitivity in intact (int) and hypophysectomized (HYPO) female (**A**) and male (**B**)rats. Baseline (BL) thermal nociception and thermal hypersensitivity are measured as paw withdrawal latency (PWL) in seconds (non-significant; 2-way ANOVA time and rat lines are factors; *n=5–7*). Incision-induced mechanical hypersensitivity in Int and HYPO female (**C**) and male(**D**) rats. BL mechanical nociception and mechanical hypersensitivity are measured as paw withdrawal threshold in grams (non-significant; 2-way ANOVA time and rat lines are factors; *n=5–7*). Post-incision time points are indicated below X-axis. Rat lines and sex are noted. Intact and HYPO female shams are for n=5 (each).

HYPO leads to substantial reduction of serum PRL levels in females (≈7 fold) and moderate decrease in males (≈2 fold) (Table 1). It was suggested that remaining PRL in serum could be attributed to extra-pituitary origin [26–29]. As expected [30, 31], HYPO, which leads to substantial decrease of luteinizing hormones (LH) and to a lesser extent follicle stimulating hormone (FSH) [32, 33], reduced serum estradiol levels (Table 1). Basal levels (BL) of thermal (heat) nociception are not statistically different in HYPO versus intact (Int) male and female rats (Figure 1A and 1B). This is in accordance with previously published data [34]. Incision-induced thermal hypersensitivity was similar for HYPO compared to intact females (in estrous phase) at all tested post-surgery time points (Figure 1A). HYPO did not significantly change postoperative thermal hypersensitivity at 1, 3 and 7 days post-surgery for male rats (Figure 1B). Like intact females and males, HYPO females and males exhibited statistically comparable levels and development time-course of postoperative thermal hypersensitivity. Thus, thermal hypersensitivity for HYPO and intact rats of both sexes was resolved by 7 days post-incision.

We next evaluated whether postoperative mechanical hypersensitivity was altered in male and female rats that have undergone hypophysectomy. Basal (BL) behavioral responses to mechanical stimuli were approximately equal in HYPO versus intact female as well as male rats (Figure 1C and 1D). At 24 hours post-surgery, postoperative mechanical hypersensitivity was slightly higher but not statistically significant (lower threshold) in HYPO female versus HYPO male rats (13.83 ± 2.7 for females *vs*.19 ± 2.5 for male; 3-way ANOVA with sex, rat line and time as factors; p=0.15, n=6). However, removal of pituitary in females and males did not influence the level and time-course of postoperative mechanical hypersensitivity at 1, 3 and 7 days post-surgery (Figure 1C and 1D). In summary, incision-induced thermal and mechanical hypersensitivity levels and development time-course were not affected by HYPO in female and male rats.

### Ovariectomy and postoperative hypersensitivity

**Figure 2:**
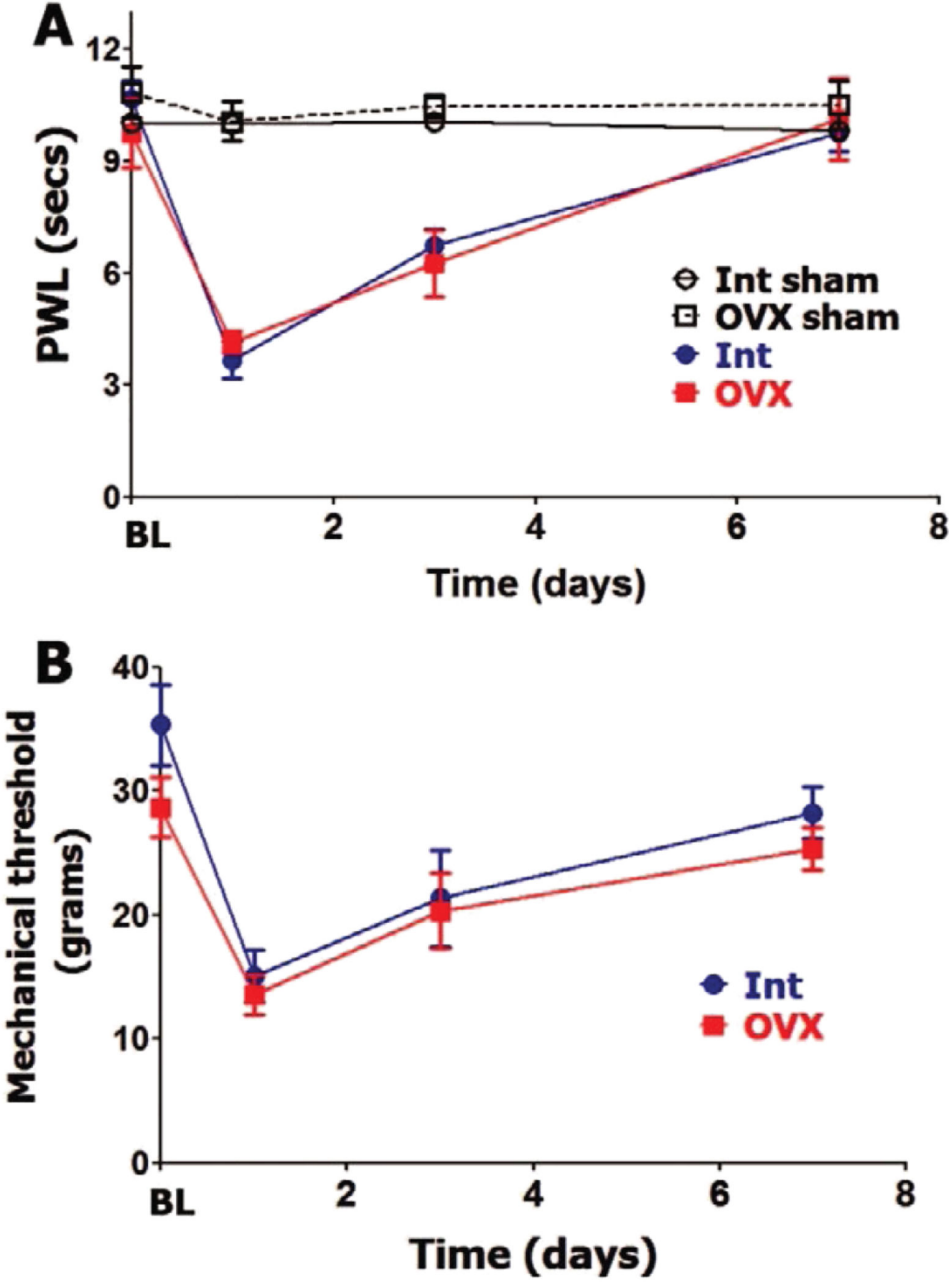
Ovariectomy and postoperative hypersensitivity in female rats Incision-induced thermal (**A**) and mechanical (**B**) hypersensitivity are evaluated in Int female rats in estrous phase and ovariectomized (OVX) female rats. BL nociception and hypersensitivity are measured as PWL for thermal stimuli and threshold for mechanical stimuli (non-significant; 2-way ANOVA time and rat lines are factors; *n=5–6*). Post-incision time points are indicated below X-axis. Rat lines are noted. Int and OVX shams are for n=5 (each).

Since hypophysectomy substantially decreases gonadal hormone production, we separately evaluated roles of gonad-produced hormones on postoperative hypersensitivity in female and male rats. OVX lowers estradiol levels three-fold and testosterone to levels that are not detectable (Table 1). Besides gonadal hormone, OVX results in reduction of some pituitary hormones, such as serum PRL [35] (Table 1). Baseline (BL) thermal and mechanical nociception was not affected by OVX (Figure 2A and 2B). OVX rats did not differ from intact female rats (in estrous phase) in levels of thermal (Figure 2A) and mechanical (Figure 2B)postoperative hypersensitivity. For both OVX and intact female rats, postoperative hypersensitivity receded to almost pre-operated levels at 7 days post-surgery (Figure 2A and 2B).

### Gonadectomy and postoperative hypersensitivity

**Figure 3:**
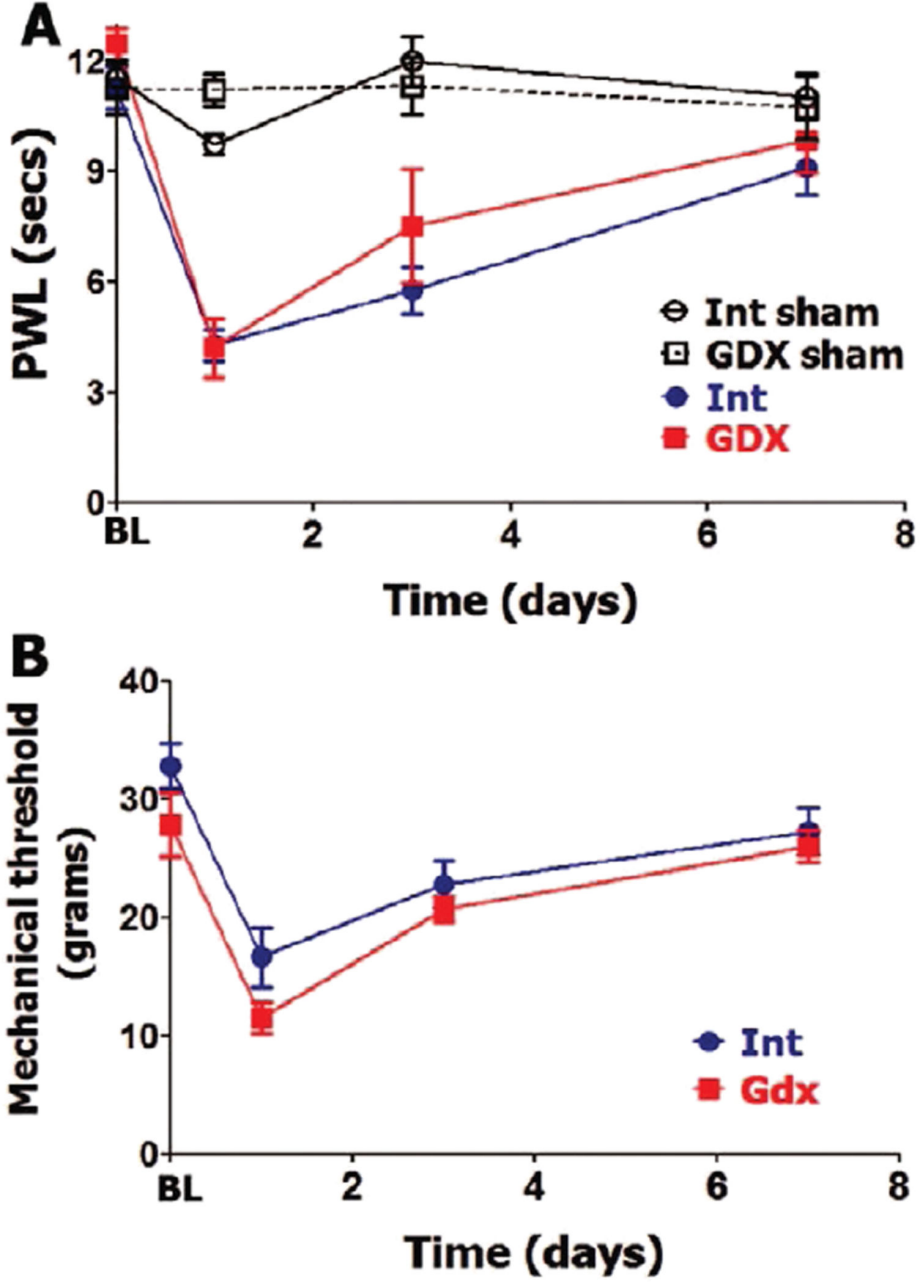
Gonadectomy and postoperative hypersensitivity in male rats Incision-induced thermal (**A**) and mechanical (**B**) hypersensitivity are evaluated in Int and gonadectomized (GDX) male rats. BL nociception and hypersensitivity are measured as PWL for thermal stimuli and threshold for mechanical stimuli (non-significant; 2-way ANOVA time and rat lines are factors; *n=5–6*). Post-incision time points are indicated below X-axis. Rat lines are noted. Int and GDX shams are for n=4 (each).

GDX rats were found to have undetectable amounts of serum total testosterone and a reduction of serum estradiol (≈1.5 folds) and PRL compared to intact males (Table 1). GDX intervention in male rats did not affect BL thermal and mechanical nociception (Figure 3A and 3B). Incision-induced thermal hypersensitivity lasted 7 days in intact and GDX males, and was statistically insignificantly different at every evaluated post-surgery time point (Figure 3A). Similarly, GDX and intact male rats have almost equal mechanical hypersensitivity and development time-course. Overall, OVX on female and GDX on male rats did not affect level and time-course of postoperative thermal or mechanical hypersensitivity.

## Discussion

Surgical intervention activates the endocrine system [1–3]. This activation is known to affect the HPA axis, altering pituitary and gonadal hormone production after many different surgical procedures [1,6–8]. The roles of pituitary hormones in modulation of postoperative hypersensitivity/pain are unknown. Here, we investigated influence of pituitary hormones on postoperative hypersensitivity levels and development time-courses. Since the disruption of pituitary hormone production dramatically reduced gonadal hormone levels, we also examined the influence of hormones generated by gonads on postoperative hypersensitivity in female and male rats. Pituitary and gonadal hormone supply were ablated by disconnection or removal of the pituitary (*i.e*., Hypophysectomy), ovaries (*i.e*., ovariectomy) or testis (*i.e*.,gonadectomy). Our results indicate that HYPO, OVX and GDX procedures on female and male rats do not significantly influence thresholds and development time-courses of thermal and mechanical postoperative hypersensitivity. HYPO females compared to HYPO males also had statistically in significant differences in level and development of postoperative hypersensitivity. Finally, as expected [20, 36], intact male and female rats have comparable postoperative hypersensitivity throughout 7 post-surgery days; at this time point, postoperative hypersensitivity had declined to almost baseline nociception.

The role of HYPO on pain levels and development dynamics has not been widely studied in different animal pain models or different human pain conditions. Hence, there is no unified theory or known underlying mechanism in regards to the contribution of the pituitary gland to pathological pain/hypersensitivity. Scarce information exists on the influence of pituitary hormones on pain in certain conditions. Clinical studies have provided some information though, as HYPO has traditionally been successfully used to suppress severe pain in some types of cancer [37–41]. It is thought that some supratentorial intracranial lesions, including HYPO, may effectively control chronic but not acute pain in humans [42]. Thus, neural-endocrine circuits for acute pain models could be accounted mostly, if not all, to the sympathetic system [43]. However, this paradigm may not apply to all acute pain models, as HYPO can produce analgesia in formalin-induced tonic pain model [44]. Additionally, HYPO can almost completely abolish the stress-induced analgesia found in some acute pain models [45–47]. Like HYPO, ablation of gonadal hormones affects acute/tonic pain in the formalin model [48]. Overall, effects of ovariectomy and gonadectomy on hypersensitivity levels in acute pain conditions are similar to removal of pituitary. However, circulating gonadal hormones substantially influence responses to systemic morphine [49]. This is understandable since opioids show significant sex-based differences for analgesic efficacy, safety profile and abuse/addiction potential [50–53].

Ablation of pituitary or gonadal hormone supply does not significantly influence postoperative thermal or mechanical hypersensitivity in an acute post-surgery pain model. However, recent reports have shown that surgical procedures can trigger up-regulation of traditional pituitary hormones in extra-pituitary levels at the surgery affected tissues and spinal cord that are in the vicinity of peripheral and central terminals of sensory neurons [20,54].

Gonadal hormones can also be supplied by extra-gonadal sources [55]. Our results imply that despite activation of the HPA axis by surgical interventions, extra-pituitary and extra-gonadal origins of these hormones may play a key role in regulation of postoperative pain. Regulation of extra-pituitary and extra-gonadal hormone production following surgery is a seldom studied area. Nevertheless, this could be important area of research, since certain pituitary and gonadal hormones could be vital contributors in conditions where surgery is accompanied by prolonged stress that strongly influences the time-course of postoperative hypersensitivity in rodents [56,57].

## Figures and Tables

**Table 1:** Pituitary and gonadal hormones in serum of intact, hypophysectomized, ovariectomized and gonadectomized rats.

	Estradiol (pg/ml)	Testosterone (ng/ml)	PRL (ng/ml)
Female	47±7	0.05±0.01	75±8
Male	22±6	7±2	14±3
Hypophysectomized female	14±2	NE	11±3
Hypophysectomized male	12±3	NE	8±2
Ovariectomized	13±4	ND (<0.01)	9±2
Gonadectomized	17±4	ND (<0.01)	10±3

NE – not evaluated; ND – non-detectable levels; N=4–6
